# Primate Lentiviral Vpx Commandeers DDB1 to Counteract a Macrophage Restriction

**DOI:** 10.1371/journal.ppat.1000057

**Published:** 2008-05-02

**Authors:** Natalia Sharova, Yuanfei Wu, Xiaonan Zhu, Ruzena Stranska, Rajnish Kaushik, Mark Sharkey, Mario Stevenson

**Affiliations:** Program in Molecular Medicine, University of Massachusetts Medical School, Worcester, Massachusetts, United States of America; King's College London, United Kingdom

## Abstract

Primate lentiviruses encode four “accessory proteins” including Vif, Vpu, Nef, and Vpr/Vpx. Vif and Vpu counteract the antiviral effects of cellular restrictions to early and late steps in the viral replication cycle. We present evidence that the Vpx proteins of HIV-2/SIV_SM_ promote virus infection by antagonizing an antiviral restriction in macrophages. Fusion of macrophages in which Vpx was essential for virus infection, with COS cells in which Vpx was dispensable for virus infection, generated heterokaryons that supported infection by wild-type SIV but not Vpx-deleted SIV. The restriction potently antagonized infection of macrophages by HIV-1, and expression of Vpx in macrophages *in trans* overcame the restriction to HIV-1 and SIV infection. Vpx was ubiquitylated and both ubiquitylation and the proteasome regulated the activity of Vpx. The ability of Vpx to counteract the restriction to HIV-1 and SIV infection was dependent upon the HIV-1 Vpr interacting protein, damaged DNA binding protein 1 (DDB1), and DDB1 partially substituted for Vpx when fused to Vpr. Our results indicate that macrophage harbor a potent antiviral restriction and that primate lentiviruses have evolved Vpx to counteract this restriction.

## Author Summary

Defense against infection by the primate lentiviruses HIV/SIV is mediated primarily by antibodies that can neutralize the virus and by cytotoxic cells that can recognize and kill other virus-infected cells. However, in the past several years, research has revealed the existence of an additional line of host defense against HIV/SIV. It is now apparent that cells contain factors (also known as cellular restrictions) that potently inhibit virus infection. This has forced primate lentiviruses to evolve a strategy to counteract these cellular restriction factors. For example, HIV/SIV encode an accessory protein called Vif, whose function is to neutralize a cellular restriction to HIV/SIV infection. Our study provides evidence for a novel restriction that is expressed by macrophages and which potently antagonizes HIV and SIV infection. We describe how the virus protects itself from this cellular restriction. The goal is to harness this cellular restriction as the basis for a novel therapeutic strategy against HIV infection.

## Introduction

The genomes of primate and non-primate lentiviruses encode “accessory” proteins from short open reading frames which are absent from the genomes of simple retroviruses [1]. The function of two of the accessory proteins, the Vif and Vpu proteins, have been defined: Vif antagonizes the antiviral activity of cellular Apobec 3 cytidine deaminases [2] and Vpu antagonizes the activity of tetherin to promote release of virions from the cell surface [3]. In all HIV and SIV lineages, the central viral region (overlapping Vif and Tat open reading frames) encodes at least one gene which is usually termed viral protein R (Vpr). Members of the HIV-2/SIV_SM_/SIV_MAC_ lineage contain an additional gene in this region termed viral protein X (Vpx) which was originally derived from the African green monkey *vpr* gene by an ancestral recombination event [4]. Both Vpr and Vpx proteins are packaged into virions through association with the Gag polyprotein [5]–[7] and this points to an early role for these proteins in the virus life cycle (i.e., at a point proceeding *de novo* production of viral proteins). Most of the information regarding the roles of Vpr and Vpx proteins in primate lentivirus replication has been derived from studies with HIV-1 Vpr. The Vpr protein of HIV-1 has been shown to promote the accumulation of cells in the G2 stage of the cell cycle [8]–[11] and to associate with the DNA repair enzyme Uracil DNA glycosylase [12]. In addition, Vpr has been shown to promote the infection of terminally differentiated macrophages and dendritic cells [13]–[17]. These HIV-1 Vpr-ascribed activities segregate between the Vpx and Vpr proteins of HIV-2/SIV_SM_: Vpr of HIV-2/SIV_SM_ induces cell cycle arrest and associates with UDG but is dispensable for macrophage infection while Vpx neither induces cell cycle arrest nor associates with UDG [4],[18]. However, Vpx is essential for infection of simian macrophages by SIV in vitro and following infection of simian macrophages by Vpx minus SIV_SM_, late cDNA product are reduced while 2-LTR cDNAs, which are formed only after completion of reverse transcription, are absent [4],[18]. Whether any of these activities relate to the functional role of Vpr/Vpx proteins in primate lentivirus replication, is unclear. In order to understand the functions of the Vpr/Vpx proteins in macrophage infection, we have focused on Vpx because of its profound impact on macrophage infection. In addition, its effect can be studied independently of other Vpr/Vpx-assigned activities including UDG association and cell cycle arrest.

## Results

### Vpx is required for infection of heterokaryons between permissive and non-permissive cells

We previously demonstrated that Vpx of HIV-2/SIV_SM_ was essential for early events in macrophage infection yet dispensable for infection of CD4 lymphocytes [4]. We studied Vpx function in the context of SIV_SM_ PB j which represents a primary isolate [19]. To increase particle infectivity and facilitate analysis of early events in the viral life cycle, viruses were pseudotyped with VSV-G envelope proteins. Although VSV pseudotyping has been shown to alleviate the defects exhibited by other accessory gene mutants such as Nef, pseudotyping did not alleviate the infectivity defect of Vpx-deleted viruses in macrophages. In order to gauge infection of primary macrophages under single cycle conditions, we quantitated viral cDNAs (mainly 2-LTR cDNA) by real time PCR. In this study, where we were dealing with a restriction and the viral target of the restriction was unknown, it seemed prudent to conduct experiments predominantly with viruses intact for all open reading frames as opposed to recombinant indicator viruses.

**Figure 1:**
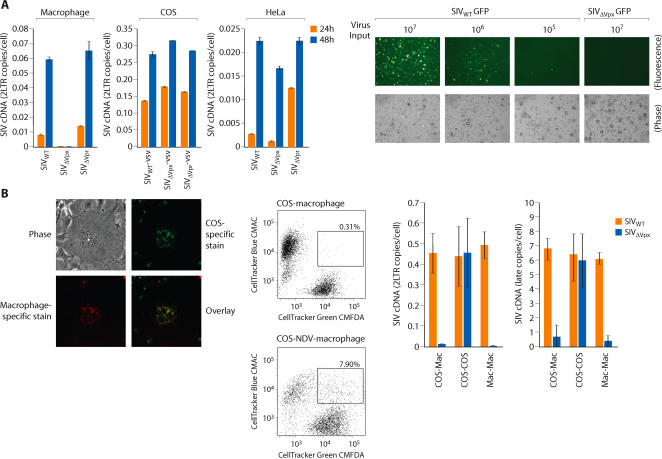
Vpx antagonizes an antiviral restriction in macrophages. (A) Differential susceptibility of macrophages and cell lines to infection by wild type and Vpx-deleted (ΔVpx) or Vpr-deleted (ΔVpr) variants of SIV_SM_. Virus infection was gauged from the levels of viral 2-LTR cDNA at 24 and 48 h post infection. Right panels, comparison of infectivity of wild type and Vpx deleted SIV GFP variants. Macrophages were infected with 10^7^, 10^6^ or 10^5^ RT unites of SIV_WT_ -GFP or 10^7^ RT units of SIV_ΔVpx_ GFP. Macrophages were visualized 48 h post infection by phase and fluorescence microscopy (B) Differential infectivity of wild type and Vpx-deleted SIV variants for macrophage-COS heterokaryons. Heterokaryons were formed between primary macrophages and between COS cells expressing fusogenic HN and F proteins of Newcastle Disease Virus. To visualize heterokaryons by fluorescence microscopy, COS cells were stained with DiO (green) and macrophages were stained with DiI (red) (magnification ×320; left panel). FACS analysis of macrophage-COS heterokaryons (middle panel). COS cells were cotransfected with NDV HN and F expression vectors (COS-NDV) or with empty, control vectors (COS). COS cells were stained with CellTracker Green CMFDA and macrophages were stained with CellTracker Blue CMAC. Double-stained cells were sorted as indicated by the gate. Susceptibility of macrophage/COS heterokaryons and COS and macrophage homokaryons to infection by SIV_WT_ and SIV_Δvpx_ virus variants (right panel). Infection was gauged from levels of late cDNAs and 2-LTR circle cDNAs (error bars are s.d. of 6 replicate samples from two independent experiments).

The profound requirement for Vpx in macrophage infection by HIV-2/SIV_SM_ is illustrated in **Figure 1A**. 2-LTR cDNA is formed only after completion of viral reverse transcription and translocation of viral cDNA to the nucleus where circularization occurs. Levels of 2-LTR cDNA in macrophages infected with a wild-type SIV and an SIV variant lacking Vpr were indistinguishable (**Figure 1A**). In contrast, viral 2-LTR cDNA was reduced at least 100 fold in macrophages infected with an SIV variant lacking Vpx (**Figure 1A**). In COS cells and in HeLa cells, viral cDNA synthesis with wild type and Vpr-deleted or Vpx-deleted viruses were similar. Although 2-LTR cDNA was not detected in macrophages infected with SIVΔ Vpx, late viral cDNAs were evident but at a reduced level. Late cDNAs were reduced 15 fold and 2 fold at 24 and 48 h respectively in SIVΔ Vpx as compared to wild type infection of macrophages (**Figure S1**). Our original study [4] on the requirement for Vpx in SIV infection of monkey macrophages reported a predominant defect in 2-LTR circle formation and an approximately 3 fold defect in late cDNA synthesis using non quantitative PCR. This is consistent with the defect observed in this study which involves infection of human macrophages with SIV. The greater than 100 fold defect in 2-LTR cDNA formation was recapitulated in macrophage infections with Vpx deleted SIV variants expressing GFP (**Figure 1A**). This analysis revealed that an SIV variant lacking Vpx was at least 100 fold less infectious than the wild type counterpart (**Figure 1A**). Although Vpx was necessary for macrophage infection, it was dispensable for infection of COS/HeLa cells (**Figure 1A**)**.** This suggested the existence of cellular activities, differentially expressed between macrophages and COS or HeLa cells, which impact primate lentivirus infection. One possibility was that COS and HeLa cells contain a cellular activity which promotes virus infection but in macrophages, this activity must be activated by the Vpx protein. An alternative possibility was that macrophages contain a cellular restriction to infection which is counteracted by the Vpx protein and this cellular restriction is not expressed in COS or HeLa cells. To distinguish between these two possibilities, we used a strategy previously adopted to characterize the mechanism by which Vif promotes viral infection [20],[21]. Heterokaryons were generated between macrophages and COS cells and the susceptibility of the heterokaryons to infection by SIV_WT_ and SIVΔ Vpx was compared. When the fusogenic proteins of Newcastle Disease Virus (NDV) were expressed in COS cells, these cells readily underwent fusion with primary macrophages (**Figure 1B**). Macrophage/COS heterokaryons (double staining cells) were isolated by fluorescence-activated cell sorting (FACS). Double staining cells were not observed when normal COS cells (not expressing NDV proteins) were mixed with macrophages (**Figure 1B**). As an additional control, macrophage homokaryons were produced using polyethylene glycol (PEG). Both macrophage/COS heterokaryons as well as COS and macrophage homokaryons were infectible by wild type SIV (**Figure 1B**). In contrast, macrophage homokaryons and macrophage/COS heterokaryons were resistant to SIVΔ Vpx infection (**Figure 1B**). Since fusion with COS cells did not relieve the block to macrophage infection by SIVΔ Vpx, this indicated that macrophages harbor an antiviral restriction which is counteracted by the Vpx protein and this restriction is absent from COS and HeLa cells.

### Vpx counters the restriction *in trans*

**Figure 2:**
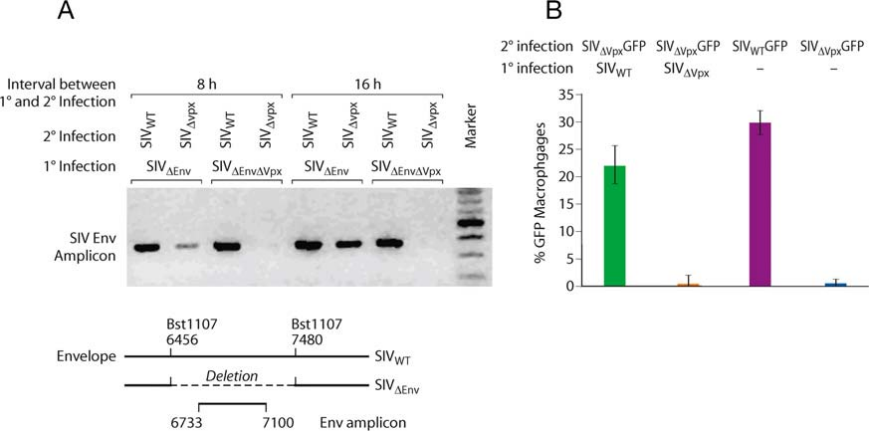
Vpx delivered to macrophages by SIV_WT_ infection removes a block to subsequent infection by SIV_Δvpx_. (A) Macrophages were initially infected (1° infection) with envelope deleted SIV variants harboring intact or defective Vpx genes. The nature of the envelope deletion is shown in the lower panel. Those cells were then super-infected (2° infection) with SIV_WT_ or SIV_Δvpx_ variants harboring intact envelope genes. cDNA products resulting from the super infection were then specifically amplified using envelope-specific primers. (B) Similar experiment was performed using SIV_WT_ or SIV_Δvpx_ for 1° infection and SIV_WT_ or SIV_Δvpx_ GFP variants for 2° infection. Number of GFP-positive cells was determined 24 hr post 2° infection (error bars are s.d., n = 3).

Vpx and Vpr are virion proteins and would thus be predicted to exert their function in the target cell shortly after infection and prior to *de novo* synthesis of viral proteins. Therefore, we examined whether Vpx delivered to macrophages would alleviate the restriction *in trans* to subsequent infection by a Vpx deleted virus. Macrophages were first infected (1° infection) with envelope deleted SIV variants harboring intact or defective Vpx genes **(** **Figure 2** **)**. After an additional 8 or 16 hours, macrophages were subsequently super-infected (2° infection) with wild type or Δ Vpx SIV variants. Viral cDNA products were amplified using envelope-specific PCR primers **(** **Figure 2** **)**. cDNA products amplified by these envelope specific primers were derived specifically from the secondary (2°) infection since viruses used in the primary infection (1°) lacked an intact envelope gene **(** **Figure 2** **)**. Infection of macrophages harboring a wild type Vpx gene alleviated the block to subsequent SIVΔ Vpx super-infection 8 or 16 hours later **(** **Figure 2** **)**. In contrast, macrophages initially infected with a ΔVpx SIV remained refractory to subsequent super-infection **(** **Figure 2** **)**. Infection of macrophages with SIV_WT_ also removed the restriction to subsequent infection by a Vpx minus SIV variant expressing GFP (**Figure 2**). This provided evidence that Vpx, delivered to the target cell, can counteract the restriction *in trans*.

### Role of ubiquitylation in biological activity of Vpx

**Figure 3:**
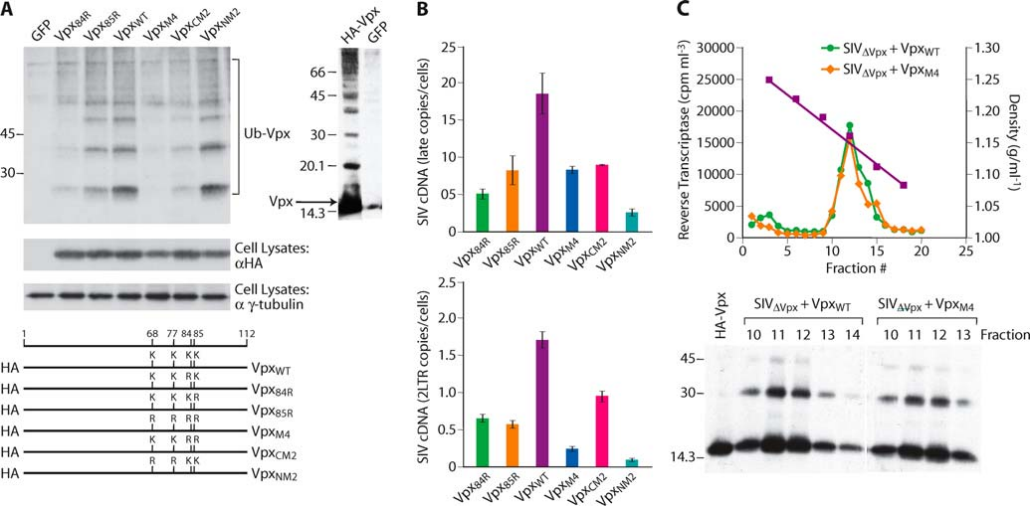
Role of the proteasome ubiquitylation system in regulation of SIV infectivity by Vpx. (A) Identification of ubiquitylated residues in Vpx. Wild type and lysine mutant Vpx proteins (HA tag) were expressed in 293T cells expressing Histidine-tagged ubiquitin. (B) Susceptibility of primary macrophages to infection by SIV_Δvpx_ packaging either wild type Vpx or lysine substitution mutants of Vpx. Vpx proteins were packaged after co-transfection of SIV_Δvpx_ proviral DNA with plasmids expressing wild or lysine mutant Vpx proteins or GFP as a control. Virus infection was gauged from quantitation of late viral cDNAs and 2-LTR cDNAs 48 h post infection (error bars are s.d. of 3 replicate measures of a single DNA sample). (C) Packaging of wild type and non-ubiquitylated Vpx proteins in virus particles (upper panel). The presence of Vpx in gradient purified virions was determined by Western blotting with an HA antibody (lower panel).

Primate lentiviruses have evolved the accessory protein Vif to counteract the antiviral activity of cellular Apobec 3 cytidine deaminases [22]. Vif achieves this by promoting ubiquitylation and proteasomal destruction of Apobec 3 proteins [23]. To evaluate a possible role for the ubiquitin-proteasome system in the activity of Vpx, we first evaluated whether Vpx itself was ubiquitylated. HA-tagged Vpx and mutants thereof (**Figure 3A** **, lower panel**) were co-expressed in 293T cells with 6-Histidine-myc-tagged ubiquitin. Mono and poly ubiquitylated Vpx proteins were purified on nickel beads and Western blotted. Immunoblotting with an HA antibody revealed the presence of mono and poly ubiquitylated forms of Vpx (Ub-Vpx, **Figure 3A**). We also examined whether Vpx was ubiquinated by endogenous ubiquitin (as opposed to over expressed and tagged ubiquitin). HA-tagged Vpx was expressed in 293 T cells and cell lysates were directly Western blotted and probed with an HA antibody. This revealed the presence of higher molecular weight ubiquitylated forms of Vpx (**Figure 3A** **, right panel**). The extent of Vpx ubiquitylation was reduced to various extents in Vpx mutants containing single or multiple lysine to arginine substitutions (**Figure 3A**). Despite mutagenesis of all four lysines in Vpx, polyubiquitylated forms of the protein were still evident (compare GFP signal with Vpx_M4_ signal). This suggested an involvement of both lysine and nonlysine residues in Vpx ubiquitylation [24],[25]. The ability of the Vpx lysine mutants to support SIV infection of macrophages was next examined. The various mutants were packaged within SIV_ΔVpx_ virions and single cycle infection of macrophages was evaluated from synthesis of late viral cDNAs and 2-LTR cDNAs (**Figure 3B**). The infectivity of the Vpx lysine mutants was impaired to various degrees (**Figure 3B**). The Vpx mutated lacking all four lysine (Vpx_M4_) exhibited the lowest infectivity for macrophage. However, a mutant lacking the two N-terminal lysines (Vpx_NM2_) appeared to be efficiently ubiquitylated yet this mutant also exhibited a significant infectivity defect (**Figure 3B**). However, due to technical obstacles in transfection of primary macrophages, we were unable to evaluate the extent of Vpx ubiquitylation of the various lysine mutants in primary macrophages and for this reason, we were unable to directly assess whether the extent of Vpx ubiquitylation was proportional to Vpx biological activity. For subsequent experiments, we focused on the use of Vpx mutant (Vpx_M4_) containing substitutions at all four lysine residues. This mutant was efficiently packaged within virus particles at levels indistinguishable from wild type Vpx (**Figure 3C**). The packaging of the Vpx lysine mutant in viral particles suggests, at the very least, that this mutant is competent for binding to the p6 domain of the viral Gag polyprotein through which packaging of Vpr and Vpx proteins is mediated [5],[6].

### Vpx activity requires a functional proteasome

**Figure 4:**
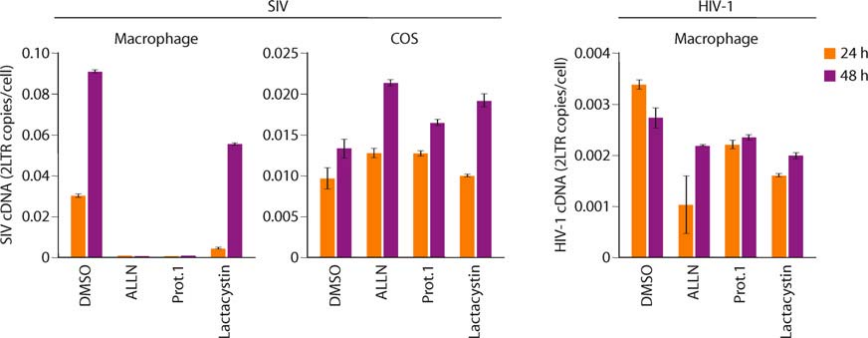
Differential impact of proteasome inhibition on SIV_WT_ and HIV-1 infection of macrophages. Effects of 3 different proteasome inhibitors on SIV infection of macrophages and COS cells and HIV-1 infection of macrophages are indicated. Viral infection (2-LTR cDNA) was gauged at 24 and 48 h post infection (error bars and s.d. of 3 replicate measures of a single DNA sample).

We next examined whether the ability of Vpx to regulate SIV infection of macrophages required proteasome function. Macrophages were treated with three different proteasome inhibitors and then infected with wild type SIV and 2-LTR cDNA was quantitated 24 and 48 hours after infection. Lactacystin had a modest yet significant effect on SIV infection and ALLN and proteasome inhibitor 1 (Prot.1) markedly impaired SIV infection of macrophages (**Figure 4**). In contrast, neither ALLN nor proteasome inhibitor 1 affected SIV infection of COS cells (**Figure 4**). Identical results were obtained for HIV-2 in that the proteasome inhibitors compromised macrophage infection but not COS cell infection (**Figure S2**). In contrast, macrophage infection by HIV-1 (which does not contain Vpx) was not compromised by the proteasome inhibitors (**Figure 4**, **right panel**). Since proteasome disruption only impacted virus infection of cells in which Vpx was required for infection, this argued that proteasome inhibition specifically impaired Vpx function rather than impacting virus infection through off-target effects. The proteasome inhibitor lactacystin exerted a more modest but significant effect on SIV and HIV-2 infection of macrophages when compared to the other proteasome inhibitors. However, we were unable to test lactacystin in primary macrophages at higher concentrations because of toxicity. Similar toxic effects of proteasome inhibitors in primary dendritic cells have also limited complete suppression of proteasome function using such inhibitors [17]. Collectively, these experiments indicate the presence of a potent antiviral restriction in macrophages that is counteracted by the Vpx protein and that the proteasome/ubiquitin system is required for the ability of Vpx to counteract this restriction.

### The macrophage restriction is active against HIV-1

**Figure 5:**
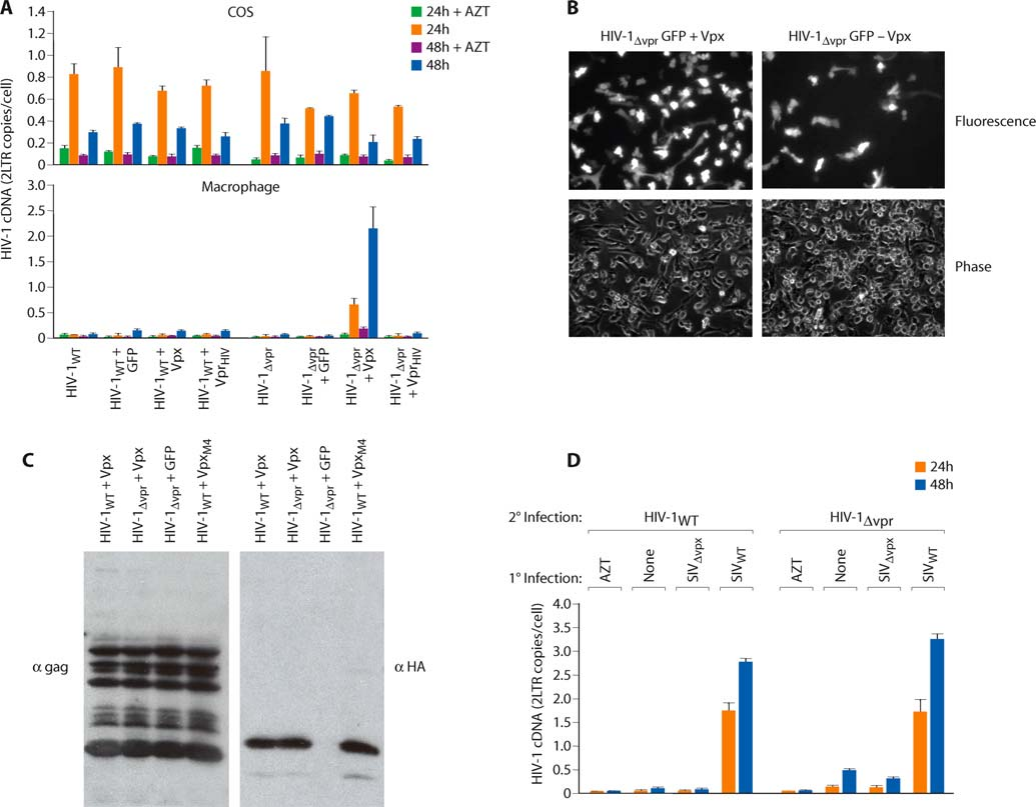
HIV-1 is sensitive to the macrophage restriction and SIV Vpx but not HIV-1 Vpr antagonizes the restriction. (A) HIV-1 Vpr and SIV Vpx proteins were packaged in HIV-1_WT_ or HIV-1_Δvpr_ viruses by cotransfection (for controls, viruses were transfected with an empty vector or a GFP-expressing vector). The infectivity of those viruses for COS (upper panel) and macrophages (lower panel) was then determined from levels of viral cDNA (2-LTR circle) at 24 and 48 h post infection. (B) Infection of macrophages with GFP-expressing HIV-1_ΔVpr_ variants in which Vpx was (+) or was not (−) packaged. GFP positive macrophages (representative fields) were visualized 48 h post infection (C) Packaging of Vpx proteins in wild type and Vpr deleted HIV-1 (D) Vpx delivered to macrophages by SIV_WT_ infection enhances the permissivity to HIV-1_WT_ and HIV-1_Δvpr_ infection. Macrophages were first infected (1° infection) with wild type or ΔVpx SIV variants, left uninfected (none) or treated with AZT. After 8 h, these cells were super-infected (2° infection) with WT or ΔVpr HIV-1 variants and HIV-1 infection (2-LTR cDNA synthesis) was determined 24 and 48 h later (error bars are s.d. of 3 replicate PCRs of a single DNA sample).

**Figure 6:**
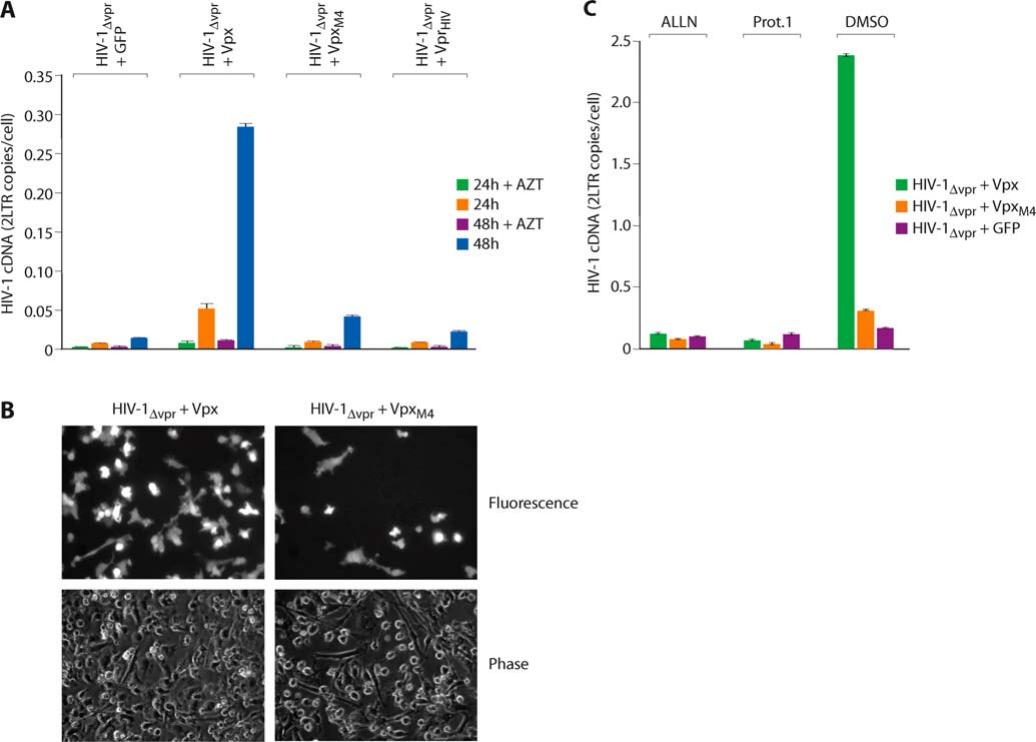
Ubiquitylation is required for enhancement of HIV-1 infectivity in macrophages. (A) Infectivity of HIV-1 variants packaging wild type or lysine-mutant Vpx proteins. Wild type HIV-1 Vpr, SIV Vpx or lysine mutant Vpx (Vpx_M4_) proteins were packaged in HIV-1_Δvpr_ and infectivity of those viruses was assessed on macrophages. Viral cDNA synthesis was evaluated 24 and 48 h post-infection. (B) Infection of macrophages with HIV-1_ΔVpr_ GFP variants packaging wild type or mutant Vpx proteins. Cells were visualized 48 h post-infection. (C) The ability of Vpx to enhance permissivity of macrophages to HIV-1 infection requires a functional proteasome. Macrophages were treated with the proteasome inhibitors ALLN or proteasome inhibitor 1 (Prot. 1) or with DMSO as a control. Those cells were then infected with HIV-1_Δvpr_ variants which had packaged wild type or lysine mutant Vpx proteins. The level of viral infection (2-LTR cDNA) was determined 24 h post-infection by PCR (error bars are s.d. of 3 replicate PCRs of a single DNA sample.)

We next evaluated whether the antiviral restriction which antagonized HIV-2/SIV_SM_ infection of macrophages was active against HIV-1. We first examined whether the Vpx protein, when packaged *in trans* within HIV-1 virions, enhanced virus infectivity for primary macrophages. While Vpx had no significant effect on the infectivity of wild type HIV-1, the infectivity of HIV-1Δ Vpr for macrophages was profoundly enhanced by Vpx but not by HIV-1 Vpr (**Figure 5A** **, lower panel**). The infectivity enhancement was also apparent in macrophages infected with an HIV-1 variant expressing green fluorescent protein (GFP) (**Figure 5B**). Thus, while HIV-1 was infectious for macrophages, its ability to infect these cells was markedly enhanced in the presence of Vpx. Vpx had no effect on the infectivity of wild type or ΔVpr HIV-1 for COS cells (**Figure 5A** **, upper panel**). A possible explanation for the ability of Vpx to compliment HIV-1 ΔVpr but not wild-type HIV-1 is that Vpx and HIV-1 Vpr proteins compete for packaging within HIV-1 virions. An alternative possibility was that these proteins do not compete for packaging into virions but compete for interaction with the restriction after infection has occurred. Western blotting analysis revealed that both wild type and lysine mutant (Vpx_M4_) Vpx proteins were packaged into wild type and Vpr deleted HIV-1 virions (**Figure 5C**). This suggested that HIV-1 Vpr competed with Vpx in the target cell following infection and this competition precluded the ability of Vpx to activate the restriction. A prediction of this is that delivery of Vpx to this target cell prior to HIV-1 infection should be sufficient to inactivate the restriction and subsequently enhance macrophage infection by both wild type and Vpr deleted HIV-1. To evaluate this, we bypassed the requirement for Vpx packaging by directly introducing Vpx into the target cell by SIV_WT_ infection. The susceptibility of those cells to infection by wild type or Vpr-deleted HIV-1 variants was then examined. In this case, the infectivity of both wild type and vpr deleted HIV-1 variants for macrophages was enhanced when Vpx was first introduced into the cell by SIV_WT_ infection (**Figure 5D**). In contrast, prior infection with a SIVΔ Vpx variant did not enhance subsequent HIV-1 infection of macrophages (**Figure 5D**). Therefore, in the absence of competition by packaged Vpr, Vpx greatly enhanced HIV-1 infectivity for macrophages. We next evaluated whether the ability of Vpx to enhance HIV-1 infectivity depended upon its ubiquitylation. As was the case for SIV, a Vpx mutant lacking ubiquitylation sites (Vpx_M4_) did not enhance HIV-1 infectivity when packaged within HIV-1Δ Vpr virions (**Figure 6A**). This was also apparent in infections using indicator viruses (**Figure 6B**). In this case, the ability of Vpx to enhance the infectivity of a Vpr deleted HIV-1 variant expressing GFP was compromised by the M4 mutation. In addition, the ability of Vpx to enhance HIV-1 infectivity required proteasome function in that Vpx failed to enhance permissiveness of macrophages to HIV-1 infection in macrophages in which proteasome function was disrupted by ALLN or proteasome inhibitor 1 (**Figure 6C**).

### Vpx function requires damaged DNA binding protein 1 (DDB1)

**Figure 7:**
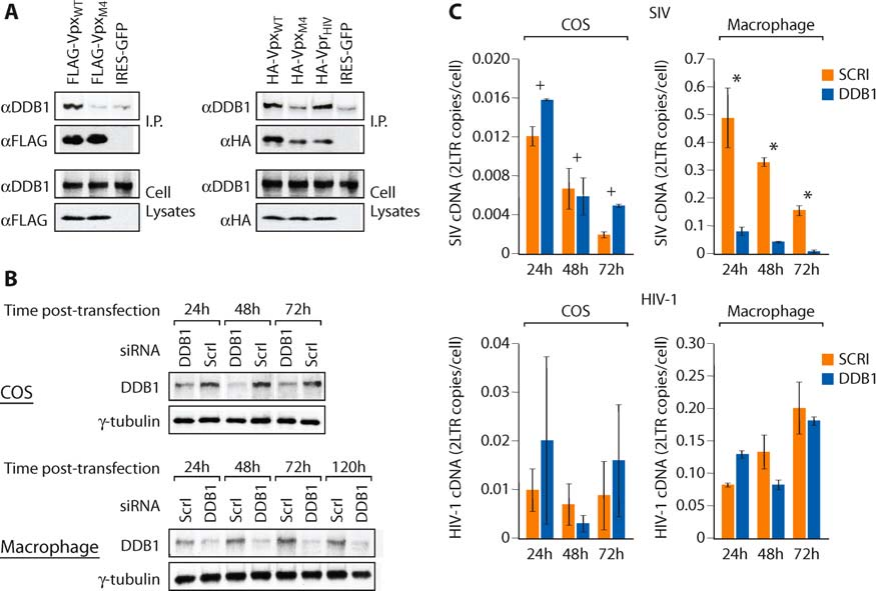
Inactivation of the macrophage restriction to SIV by Vpx requires DDB1. (A) Association of SIV Vpx with endogenous DDB1. Association of DDB1 with wild-type Vpx (Vpx_WT_) and non-ubiquitylated Vpx (Vpx_M4_) was evaluated in 293T cells expressing FLAG-tagged Vpx (left panels) or HA-tagged Vpx (right panels) or IRES-GFP as a control. FLAG and HA immunoprecipitates were immunoblotted with DDB1 or FLAG and HA antibodies (upper panels). Levels of endogenous DDB1 and expressed Vpx in cell lysates were confirmed by Western blotting with a DDB1 antibody and with FLAG/HA antibodies respectively (lower panels). (B) Efficiency of siRNA-mediated silencing of DDB1 expression in COS cells and in macrophages was evaluated by Western blotting with DDB1 antibody at the indicated intervals post siRNA-transfection (ScrΙ-scrambled siRNA control). (C) Impact of DDB1 silencing on SIV and HIV-1 infection of COS cells and macrophages. SIV and HIV-1 infection was gauged from the quantity of viral cDNA (2-LTR) at 24, 48 and 72 h post infection (+, p>0.05; *, p<0.005) (error bars are s.d. of replicate PCRs of a single DNA sample).

Recent studies have demonstrated that the ability of HIV-1 Vpr to induce cell cycle arrest requires the E3 ubiquitin ligase complex scaffolding factor, damaged DNA binding protein 1 (DDB1) [26]–[30]. Therefore, we examined whether the ability of Vpx to counteract the macrophage restriction to SIV and HIV-1 infection was DDB1-dependent. In 293T cells, endogenous DDB1 associated with a wild-type SIV Vpx protein but not with a SIV Vpx mutant lacking lysine residues (Vpx_M4_) (**Figure 7A**). A specific association of SIV Vpx with DDB1 was apparent in coimmunoprecipitation experiment with either FLAG-tagged Vpx or with HA-tagged Vpx proteins (**Figure 7A**). If DDB1 is a functional Vpx interactor, we would predict that DDB1 silencing would only impact SIV infection of macrophages in which the restriction was expressed but not in COS cells which lack the restriction. In addition, HIV-1 Vpr did not antagonize a macrophage restriction. The activity of the restriction in HIV-1 was only revealed by the ability of Vpx to enhance HIV-1 infection of macrophages. Therefore, a prediction is that DDB1 silencing should not inhibit infection of macrophages by HIV-1. DDB1 specific siRNAs efficiently reduced DDB1 expression in COS cells and in macrophages (**Figure 7B** **, left panels**). While DDB1 silencing had no significant effect on SIV infection of COS cells (p>0.05), SIV infection was significantly impaired (p<0.005) in DDB1-depleted macrophages (**Figure 7C** **, upper right panel**). In contrast, macrophage infection by HIV-1 was not affected by DDB1 silencing (**Figure 7C** **, lower right panel**). We also used an independent strategy to deplete DDB1 in macrophages to assess its role in virus infection. Similar to the results obtained with siRNA mediated DDB1 depletion, depletion of DDB1 using DDB1-specific shRNAs also specifically impaired the susceptibility of primary macrophages to SIV infection but not HIV-1 infection **(Figure S3**). Therefore, DDB1 appears to be a specific Vpx cofactor in primary macrophages.

**Figure 8:**
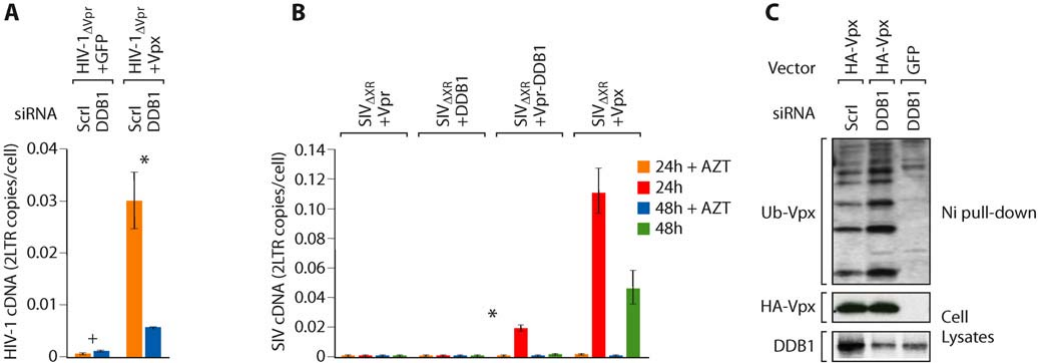
DDB1 is required for the ability of Vpx to counteract the restriction to macrophage infection by HIV-1. (A) SIV Vpx (or GFP as a control) was packaged into HIV-1_ΔVpr_ virions as described in Figure 5. Infectivity of those viruses for DDB1-depleted macrophages (DDB1 siRNA) or control macrophages (ScrΙ siRNA) were evaluated from levels of viral cDNA 24 h later (+, p>0.2; *, p<0.002). (B) DDB1 packaging partially substitutes for Vpx. A Vpx/Vpr deletion mutant of SIV (SIV_ΔXR_) was co-transfected with vectors expressing SIV Vpr, SIV Vpx, DDB1 or a Vpr-DDB1 fusion. Infectivity of the resulting viruses for macrophages was evaluated from levels of SIV cDNA at 24 and 48 h post infection (*, p<0.005). (C) Impact of DDB1 silencing on Vpx ubiquitylation. 293T cells were cotransfected with DDB1 or scrambled siRNAs and with HIS-ubiquitin and HA-Vpx or IRES-GFP expression plasmids as outlined in Figure 3. Ubiquitin-conjugated proteins were nickel purified and immunoblotted for Vpx (HA). Cell lysates were directly blotted for Vpx and DDB1 proteins (lower two panels).

We next examined whether DDB1 was required for the ability of Vpx to counteract the restriction to HIV-1 infection. Since Vpx, when packaged in HIV-1 virions, enhanced macrophage infection, we examined whether Vpx enhanced HIV-1 infection in DDB1 depleted macrophages. While packaging of Vpx in HIV-1 particles markedly increased infectivity for macrophages transfected with a scrambled siRNA (**Figure 8A**) silencing of DDB1 in macrophages significantly reduced (p<0.002) the ability of Vpx to enhance HIV-1 infection (**Figure 8A**). However, DDB1 silencing had no significant effect (p>0.05) on the infectivity of HIV-1 which had not packaged Vpx (**Figure 8A**). Since SIV Vpx but not SIV Vpr was essential for macrophage infection (**Figure 1A**), we examined whether fusion of DDB1 to SIV Vpr was sufficient to allow SIV Vpr to counteract the macrophage restriction. Packaging of Vpr alone into a Vpr and Vpx deleted SIV (SIV_ΔXR_) did not permit macrophage infection. In contrast, there was a partial and significant (p<0.005) restoration of infectivity when a Vpr-DDB1 fusion was packaged relative to infectivity of virions in which the DDB1 protein was not packaged (**Figure 8C**). Although ubiquitylation was necessary for the ability of Vpx to counteract the restriction to HIV-1 and SIV infection of macrophages, DDB1 protein was not required for Vpx ubiquitylation (**Figure 8C**). Mono and poly ubiquitylated forms of Vpx were evident and apparently increased in cells in which DDB1 expression was reduced by RNA interference (**Figure 8C**). Collectively, these results suggest that DDB1 is required for the ability of Vpx to antagonize a restriction to infect macrophages by HIV-1 and SIV but that DDB1 is not required for Vpx ubiquitylation.

## Discussion

Our study suggests that the function of Vpx is to antagonize an antiviral restriction in macrophages. Vpx exhibits similarities with the Vif protein of primate lentiviruses in that inactivation of the restriction required the proteasome/ubiquitin system. A role for the proteasome/ubiquitin system is provided by our demonstration that ubiquitylation mutants of Vpx are functionally attenuated and treatment of macrophages with proteasome disrupting agents specifically reduces their susceptibility to SIV infection but not HIV-1 infection. The inhibitory effect of proteasome inhibitors on SIV infection of primary macrophages as reported in our study appears to be at odds with studies demonstrating that HIV-1 infection of cell lines is enhanced in the presence of proteasome inhibitors [31]–[35]. The majority of these studies have involved cell lines and one of these studies [31] has suggested that proteasome inhibitors enhance HIV-1 infection by inducing G2/M cell cycle arrest thereby imparting a cellular environment that is more permissive to infection. Our study used primary macrophages and since these cells are terminally differentiated and nondividing, enhancing effects of proteasome inhibitors due to cell cycle arrest would not be manifest. A comparison of our study with the study Goujon et. al. [17] demonstrates that Vpx is essential for infection of macrophage (our study) and of dendritic cells [17]. However, there are some differences in the results obtained with Vpx mutant viruses in these two systems. In the study of Goujon et al. [17], the proteasome inhibitor MG132 marginally (1–2 fold) increased viral DNA accumulation in dendritic cells in the presence of Vpx whereas in our study, proteasome inhibitors markedly inhibited infection of macrophages by SIV but not HIV-1. Since Goujon et al. [17] reported that primary human dendritic cells were highly sensitive to the toxic effects of MG132, it is possible that differences in treatment conditions that can be employed in macrophages versus dendritic cells could account for these differences. The study of Goujon et al. [17] also showed an enhancement of SIV infection in the absence of Vpx. We did not examine the effects of proteasome inhibitors on a Vpx-deleted virus in macrophages because this variant was essentially dead in these cells.

Our study implicates DDB1 as a cellular cofactor of Vpx which is necessary for the ability of Vpx to counteract the macrophage restriction. This is supported by several independent experiments. DDB1 silencing in macrophages specifically impaired their susceptibility to infection by SIV and, in addition, impaired the ability of Vpx to enhance infectivity of macrophages by HIV-1. It is not possible to conclude at present whether DDB1 association accounts, in totality, for the biological activity of Vpx. DDB1 silencing led to a 5–10 fold reduction in SIV infectivity of macrophages whereas there was a 100 fold infectivity defect imparted by deletion of Vpx. However, RNA silencing failed to completely deplete DDB1 from primary macrophages and it is possible that residual DDB1 allowed some retention of Vpx activity in these macrophages. We also present evidence that DDB1 binds to ubiquitylated Vpx and that lysine mutants of Vpx which are inefficiently ubiquitylated exhibit reduced DDB1 binding and are impaired in their ability to support SIV infection of macrophages. Using a Vpx mutant lacking lysine residues, we present evidence that Vpx ubiquitylation is important for association with DDB1 and to counteract the macrophage restriction. Although we attribute loss of Vpx function to lack of ubiquitylation and loss of DDB1 binding, we cannot rule out the possibility that loss of function of the mutant protein was due to indirect effects of the mutations on protein structure. However, at the very least, the Vpx lysine mutant is packaged within virions which suggests that it is competent for interaction with the p6 domain of the Gag polyprotein. As with DDB1 silencing, the reduction in Vpx function imparted by mutation of all four lysines in Vpx caused a no more than a 10 fold defect in Vpx function (for example, see Figure 3B; Figure 6A,B). Therefore, ubiquitylation and DDB1 association may not fully account for the biological activity of Vpx in macrophages. However, polyubiquitylated forms of Vpx were still evident in cells transfected with a Vpx mutant lacking all lysine residues (**Figure 3A**). This suggests some degree of Vpx ubiquitination on nonlysine residues [24],[25]. Identification and mutagenesis of all ubiquitination residues on Vpx will be required before the degree to which Vpx activity depends upon ubiquitination can fully be assessed. Our study also suggests that DDB1 is not required for Vpx ubiquitylation but that Vpx ubiquitylation is necessary for association with DDB1. Therefore, the loss of function observed with the Vpx lysine mutant is likely to reflect a loss in DDB1 binding. Although SIV Vpr did not counteract the macrophage restriction, fusing it to DDB1 partially conferred this ability. This suggests that the function of Vpx may be to tether DDB1 to the reverse transcription complex upon which the restriction acts. Our study also indicates that DDB1 is required for the ability of Vpx to counter the macrophage restriction to HIV-1 infection. HIV-1 Vpr did not exhibit the ability to counter the macrophage restriction. For this reason, silencing of DDB1 did not impair susceptibility of macrophages to HIV-1 infection. However, the fact that the restriction was active against HIV-1 was revealed by the demonstration that Vpx greatly increased the permissivity of macrophages to HIV-1 infection. In this situation, silencing of DDB1 inhibited the ability of Vpx to enhance macrophage infection by HIV-1. Although Vpx is a virion protein, we do not know if DDB1 itself is packaged within virions. However, since silencing of DDB1 in the target cell inhibited SIV infection, this suggests that Vpx usurps DDB1 after infection of the target cell and likely, within the context of the reverse transcription complex.

Our study also reveals a paradox with regards to the functional consequences of HIV-1 Vpr and HIV-2/SIV Vpx interaction with DDB1. DDB1 mediates the cell cycle arrest property of HIV-1 Vpr. DDB1 was also necessary for the ability of SIV Vpx to counteract the macrophage restriction. However, SIV Vpx, although able to interact with DDB1, does not induce cell cycle arrest. Furthermore, the ability of HIV-1 Vpr to interact with DDB1 does not appear sufficient to confer upon HIV-1 Vpr the ability to efficiently counteract the macrophage restriction. Therefore, there are likely to be different biological outcomes that are dictated by the nature of the interactions that HIV-1 Vpr and SIV Vpx forge with DDB1 and its associated E3 ubiquitin ligase complex components. Further insight into the mechanisms employed by HIV-1 Vpr and HIV-2/SIV_SM_ Vpx to enhance macrophage infection may be revealed once the macrophage restriction itself is identified.

## Materials and Methods

### Proviral DNAs, virus stocks and infections

The infectious molecular clone SIV_SM_ PBj1.9 was used for the majority of experiments in this study. This clone, which is representative of the HIV-2/SIV_SM_ group of viruses, was derived from short-term peripheral blood mononuclear cell (PBMC) cultures. Unlike many other HIV-2 and SIV_SM_ clones, PBj1.9 has a complete set of uninterrupted accessory genes and replicates efficiently in macrophages and represents a physiologically relevant virus strain. Mutations which abrogated the translation of Vpx and Vpr genes are as described previously [4]. HIV-GFP (a gift of Paul Clapham, University of Massachusetts Medical School) contains an EGFP gene inserted between the envelope stop codon and nef within the HIV-1_NL4-3_ backbone. GFP expressing variants of wild type and ΔVpx SIV contain an EGFP gene inserted between Bst 1107I sites within the viral envelope gene (as schematized on Figure 2). Wild type and ΔVpr HIV-1 variants were studied in the context of HIV-1_NL4-3_. For the generation of viral stocks, 293T cells were transfected with proviral DNAs (25 µg) using a modified calcium phosphate/DNA precipitation method (Stratagene). Viruses were pseudotyped with VSV envelope glycoproteins by cotransfection of proviral DNAs with a plasmid expressing the VSV envelope glycoprotein. For encapsidation of wild type and mutant Vpx and Vpr proteins, 293T cells were cotransfected with proviral DNAs and plasmids expressing Vpx and Vpr proteins. The DNA ratio for pVSV-G, proviral clones and pIRES2-EGFP-Vpx was 1∶14∶1. For encapsidation of Vpr-DDB1 fusion proteins, 293T cells were co-transfected with an SIV deltaVpx/deltaVpr proviral clone, pIRES2-EGFP Vpr-fDDB1 and pVSV-G. The DNA ratio for pVSV-G, proviral clone and DDB1 expression plasmids was 1∶14∶1. HIV-1 and SIV stocks were normalized on the basis of reverse transcriptase activity. Viral infection efficiency was gauged from synthesis of viral cDNA products at early intervals (24 and 48 h) post-infection. PCR conditions for amplification of SIV_SM_ and HIV-1 2-LTR cDNAs are as described previously [4],[36]. cDNA copy numbers were expressed on a per cell basis after quantitation of genomic DNA copy numbers using PCR and primers to the CCR5 gene [36]. Macrophages were initially infected with VSV-pseudotyped SIV variants harboring intact or defective Vpx genes. Viruses used in the initial infection additionally lacked an intact envelope open reading frame. Macrophages were then super-infected with SIV variants which harbored intact envelope genes. As a consequence, cDNA products generated specifically by the super-infecting virus could be identified. SIV cDNA products were amplified in two rounds of PCR with JumpStart™ Red_accu_Tag™ DNA polymerase (Sigma). First round products were amplified using forward (taacaggaacaccagcaccaaca) and reverse (catctgctttccctgacaa) primers. Second-round products were amplified using forward (taacaggaacaccagcaccaaca) and reverse (aagcataacctggcggtgcaca) primers.

### Gradient purification of virions

Supernatants from 293T cells transfected with infectious molecular clones were cleared of cellular debris by low-speed centrifugation (1500 *g*, 10 min) and then filtered (0.45 µm). Virions in clarified supernatants were harvested (10,000 *g*, 2 h) and resuspended in serum-free medium (500 µl). Concentrated virions were applied to a 15–60% w/v continuous sucrose gradient and virions were resolved at 200,000 *g* for 16 h. Gradient fractions (0.5 ml) were collected and virus levels in each fraction were measured by reverse transcriptase activity. Virus particles in individual gradients were pelleted and resuspended in sample buffer and the presence of encapsidated Vpx proteins was examined by Western blotting with an αHA antibody.

### Macrophages and cell lines

Peripheral blood monocytes were obtained by elutriation and counter current centrifugation and maintained 2 days in DMEM containing 10% human serum and monocyte colony stimulating factor (MCSF) (RD Systems) and for a further 5 days in medium lacking MCSF prior to use in experiment. 293T, Hela and COS cells were maintained in DMEM containing 10% FBS.

### Proteasome inhibition

Macrophages or COS cells (8×10^5^) in 24 well plates were directly infected with VSV-G-pseudotyped viruses (1×10^6^ cpm RT/ml or 1 ug p24/ml) in the presence of proteasome inhibitors including Lactacystine (10 uM), ALLN (50 uM) and Proteasome inhibitor 1 (50 uM). After 3–5 h, supernatant was removed and replaced with fresh medium containing proteasome inhibitors. After 24 and 48 h post-infection total DNA was isolated using DNAzol reagent (Invitrogen) and analyzed by real-time PCR assay for 2LTR circles.

### Cell staining

For FACS analyis, COS cells and human macrophages were stained with 3.5 µM CellTracker Green CMFDA (5-chloromethylfluorescein diacetate) and 24 µM CellTracker Blue CMAC (7-amino-4-chloromethylcoumarin), respectively. For fluorescence microscopy, COS cells and macrophages were stained with 2.5 µM DiO (3,3′-dioctadecyloxacarbo cyanine perchlorate) and 12 µM DiI (1,1′-dioctadecyl-3,3,3′,3′-tetramethylindocarbocyanine perchlorate) respectively, according to manufacturer's instructions (Molecular Probes).

### Cell fusion

Generation of macrophage homokaryons was achieved by polyethylene glycol (PEG). Briefly, labeled cells, 15×10^6^ each group, were mixed and centrifuged at 250 *g.* 50% PEG-1450 was added dropwise to the pellet and cells incubated for 2 min at 37°C with gentle mixing. 1 ml PBS was then added dropwise to the cells over 1 min, followed by 3 ml of 2% FBS/PBS over another 2 minutes. Cells were washed 3 times with 2% FBS/PBS and plated in a 100 mm culture dish (1×10^7^ cells/dish). COS-macrophage and COS-COS cell fusion was achieved using paramyxovirus hemagglutinin-neuraminidase (HN) protein and fusion (F) protein. Briefly, COS cells were transfected with pCAGGS-HN and pCAGGS-F expression vectors encoding HN and F proteins of Newcastle disease virus (gift of Prof. T. Morrison) [37]. Sixteen to twenty hours post-transfection, COS cells were stained, mixed with stained macrophages (ratio 1∶1.5) and plated in 100 mm dishes. COS homokaryons were generated at 1∶1 ratio. After overnight incubation, cells were infected with either SIV_WT_ or SIV_ΔVpx_ for 24 h. Cell sorting was performed with a FACSAria flow cytometer using the FACSDiva software (Becton Dickinson). Double-stained cells were sorted. Total DNA was isolated using DNeasy Blood and Tissue Kit (Quiagen) and analyzed by real-time PCR assay for 2LTR circles.

### Plasmids

The SIVsm Vpx and HIV-1 Vpr genes were amplified from PBj1.9 and NL4.3 proviral clones respectively, and inserted into a pIRES2-EGFP vector (BD) either with or without a N-terminal minimum HA epitope. The upstream primer for each PCR product provided a Kozak sequence. The Vpx lysine mutants (K68,77,84,85R) were generated by Quikchange XL site-directed mutagenesis (Stratagene). The DDB1 gene was amplified and subcloned from pBj-hp125 (ATCC, MBA-126) and inserted into pIRES2-EGFP as an in frame fusion with the C-terminal of SIV Vpr. A Flag epitope was added to the N-terminal of DDB1 as flanking sequences between Vpr and DDB1. As a control, a N-terminal Flag tagged DDB1 was inserted into pIRES2-EGFP.

### Analysis of Vpx ubiquitylation

293T cells were co-transfected with HA-Vpx, HA-Vpx lysine mutants or a pIRES2-EGFP empty vector and pRGB4-6His-myc-Ubiquitin at a 1∶4 ratio using lipofectamine 2000 (Invitrogen). Non-6His tagged Ubiquitin was included as a control for Ni-NTA pull-down. 36 h after transfection, the 6His-ubiquitin conjugated proteins were purified using Ni-NTA Magnetic Agarose beads (Qiagen) under native conditions [38]. Briefly, cells were lysed in detergent buffer (10 mM Tris-Hcl pH7.5, 150 mM NaCl,1% Triton X-100 and protease inhibitor cocktail) and clarified by centrifugation at 14,000 rpm for 15 min. The cell lysates were incubated with Ni-NTA beads overnight at 4°C in detergent buffer with 300 mM NaCl, 20 mM imidazole and 5 µM MG132. The beads were washed in lysis buffer and attached proteins were eluted in elution buffer (50 mM NaH_2_PO_4_, 375 mM NaCl, 1% Triton, 250 mM imidazole pH 8.0).

### Immunoblotting

Virus pellets were lysed in RIPA buffer (50 mM Tris-Hcl pH 7.5, 150 mM NaCl, 1% NP-40, 0.5% NaDoc, 0.1% SDS and protease inhibitor cocktail) lysates of transfected cells or gradient purified virions were boiled in sample buffer, resolved by SDS/PAGE and Western blotted with the following antibodies: HA-Vpx (HA, 16B12, Covance), myc-Ubiquitin (α-ubiquitin, P4G7, Covance; α-Myc 9E10, Sigma), Capsid (polyclonal, ABI), γ-tubulin (GTU-88, Sigma), Flag-Vpx (M2, F3165, Sigma), DDB1 (Goat polyclonal antibody PC718,Calbiochem).

### RNA interference of DDB1

The siRNA sequences for DDB1 silencing in macrophages, COS-1 or 293T cells were

1. siRNA1: GCAAGGACCTGCTGTTTAT
2. siRNA2: GCATGCCAGCATTGACTTA
3. siRNA3: CCTGCATCCTGGAGTATAA

The Scrambled control siRNA sequence was CAGTCGCGTTTGCGACTGG

Macrophages or COS-1 cells were transfected twice with 60 pmol each siRNA using lipofectamine 2000. 24 h after siRNA transfection, cells were infected with RT-normalized virus as indicated. The DDB1 protein knockdown levels were examined at the same time point as cDNA analysis.

For shRNA-mediated DDB1 silencing, macrophages are infected with a TRIP lentiviral vector [39] containing or lacking DDB1 hairpin sequences. 48 h after transduction with shRNA lentivirus vectors, macrophages were infected with VSV-g-pseudotyped SIV or HIV-1 and levels of viral cDNA synthesis was assessed after additional 48 h (96 h post lentivirus vector transduction).

### Vpx-DDB1 co-immunoprecipitation

293T cells were transfected with Flag-Vpx, Flag-Vpx lysine mutant (VpxM4) or pIRES2-EGFP vector. 36 h after transfection, cells were harvested and lysed in Co-IP lysis buffer (100 mM NaCl, 50 mM Tris-Hcl pH 7.5, 5 mM MgCl2, 0.5% NP-40, protease inhibitor cocktail) and incubated with Protein A and Protein G beads (Invitrogen) conjugated anti-Flag M2 antibody overnight at 4°C. The beads were washed 4 times in a more stringent wash buffer (400 mM NaCl, 50 mM Tris-Hcl pH 7.5, 5 mM MgCl2, 0.5% NP-40, protease inhibitor cocktail). And bound proteins were boiled and eluted in 2× Laemmli's SDS-sample buffer.

### Statistical Analysis

Where indicated, data was analyzed using an unpaired Students *t test. p* values of 0.05 or lower were considered significant. Statistical analysis was performed using Graph Pad Prizm 5 software.

## Supporting Information

Figure S1 Susceptibility of macrophages to infection by wild type and Vpx-deleted SIV_SM_ variants. Virus infection was gauged from the levels of late cDNA and 2-LTR cDNA products of reverse transcription at 24 and 48 h post infection.
(0.09 MB TIF)

Figure S2 Differential impact of proteasome inhibition on HIV-2_WT_ infection of macrophage and COS cells. Effects of three different proteasome inhibitors on HIV-2 infection are indicated. Viral infection (2-LTR cDNA) was gauged 24 and 48 h post infection (error bars are s.d. of 3 replicate measures of a single sample).
(0.12 MB TIF)

Figure S3 Differential impact of shRNA-mediated DDB1 silencing on infection of primary macrophages by SIV and HIV-1. (A) DDB1 expression in primary macrophages at 72 and 96 hours post infection with a lentivirus vector expressing a DDB1 shRNA. Control cells were infected with a non shRNA expressing lentivirus vector. (B) SIV cDNA and HIV-1 cDNA levels in SIV and HIV-1 infected macrophages 96 h after transduction with lentivirus vectors expressing a DDB1 shRNA or 96 h after transduction with a vector control. Infections done in the presence of AZT were used to assess the level of carry over viral DNA contamination.
(0.23 MB TIF)
