# Supplementary figures and images for: Primate Lentiviral Vpx Commandeers DDB1 to Counteract a Macrophage Restriction
Source: PLoS Pathog. 2008 May 2;4(5):e1000057. doi: 10.1371/journal.ppat.1000057 (PMC2323106; doi:10.1371/journal.ppat.1000057)

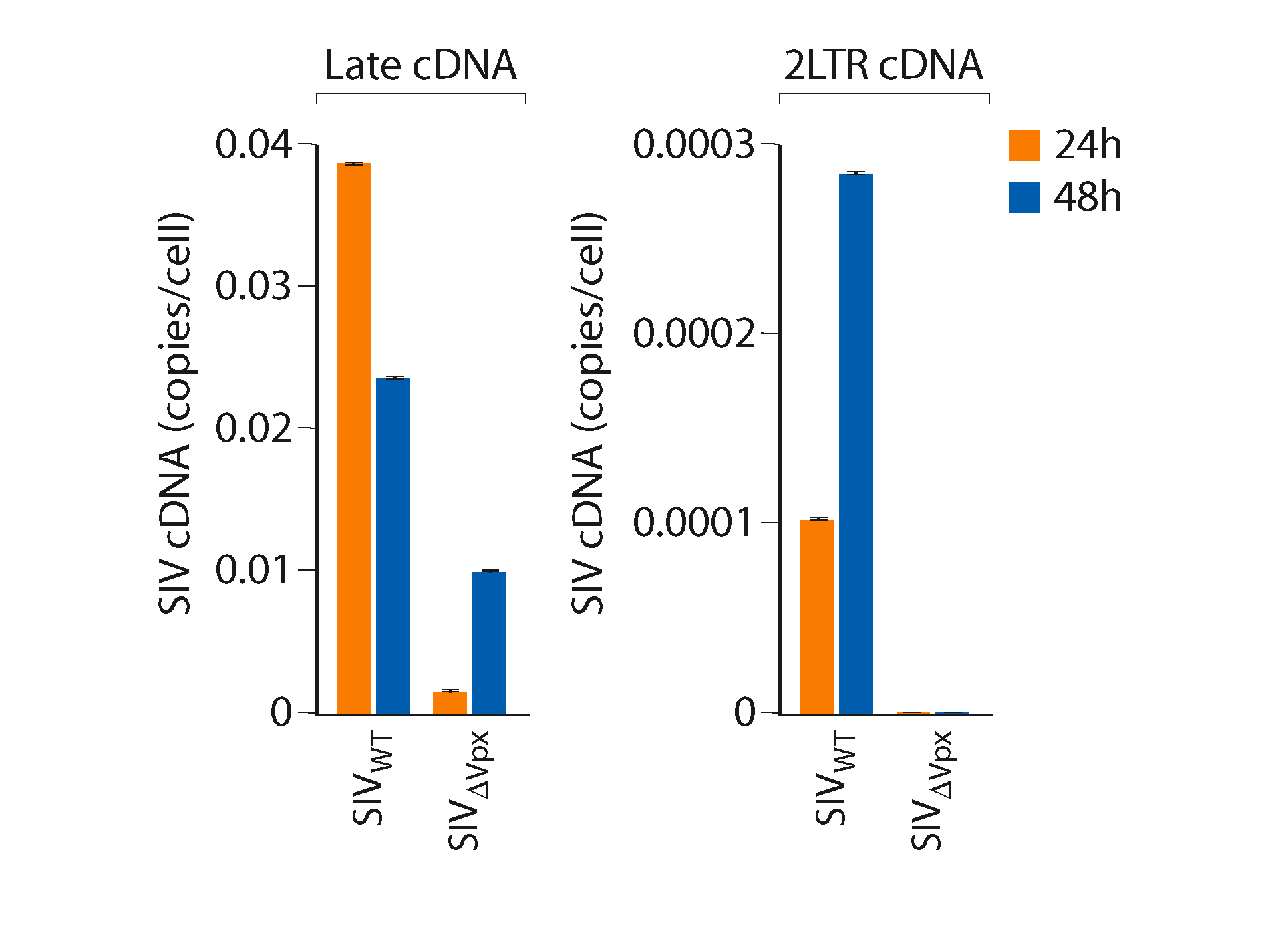

Supplement: Figure S1 — Susceptibility of macrophages to infection by wild type and Vpx-deleted SIVSM variants. Virus infection was gauged from the levels of late cDNA and 2-LTR cDNA products of reverse transcription at 24 and 48 h post infection. (0.09 MB TIF) [file ppat.1000057.s001.tif]

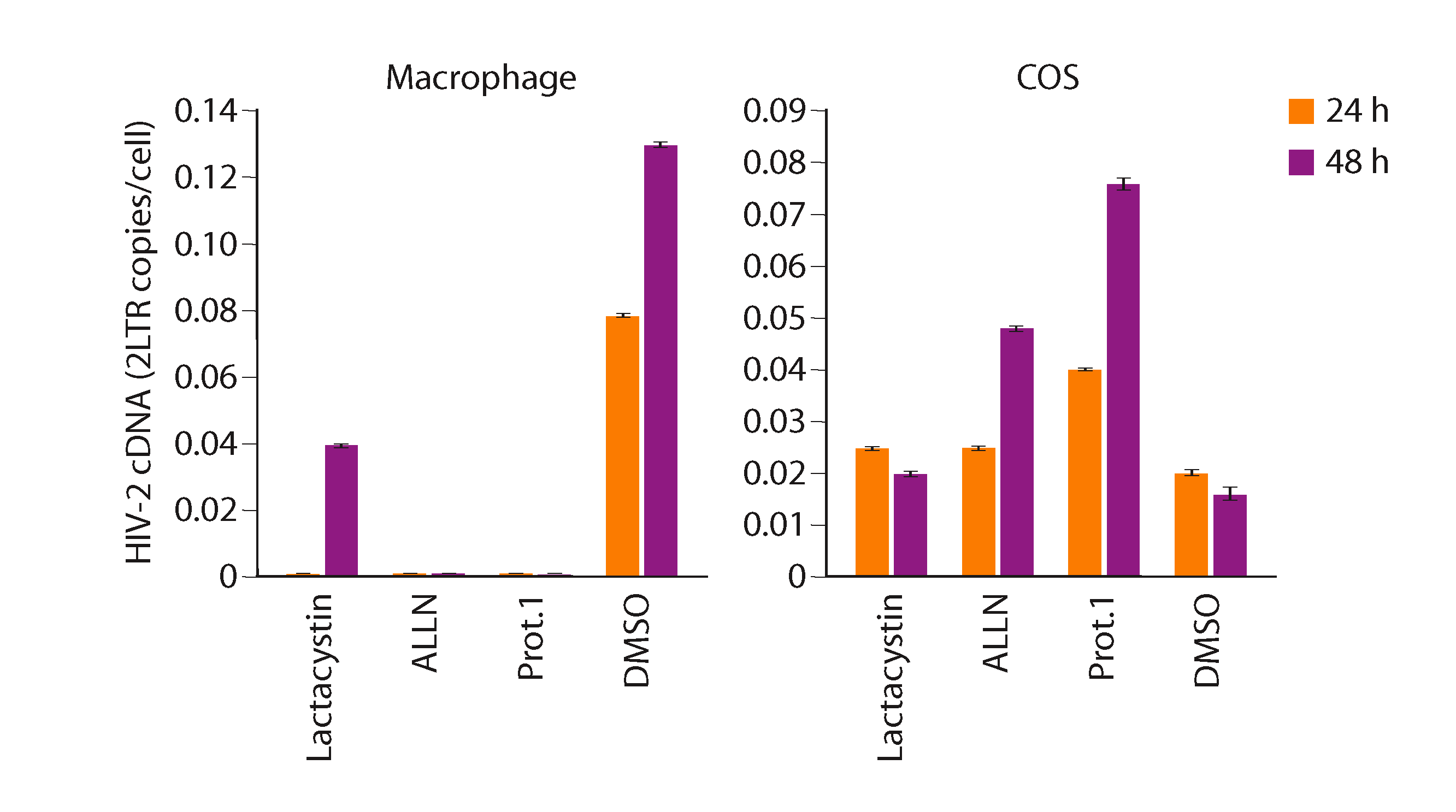

Supplement: Figure S2 — Differential impact of proteasome inhibition on HIV-2WT infection of macrophage and COS cells. Effects of three different proteasome inhibitors on HIV-2 infection are indicated. Viral infection (2-LTR cDNA) was gauged 24 and 48 h post infection (error bars are s.d. of 3 replicate measures of a single sample). (0.12 MB TIF) [file ppat.1000057.s002.tif]

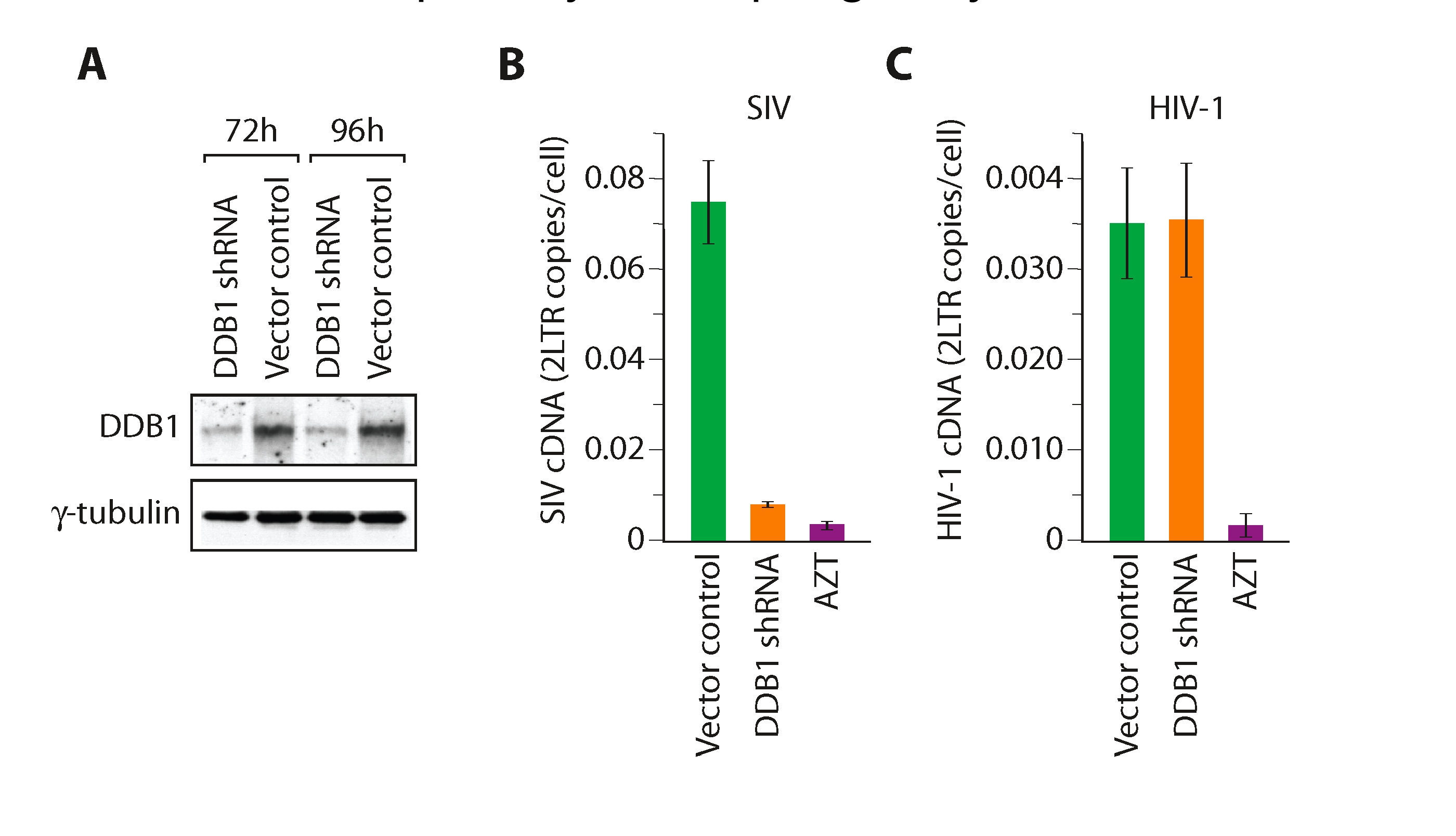

Supplement: Figure S3 — Differential impact of shRNA-mediated DDB1 silencing on infection of primary macrophages by SIV and HIV-1. (A) DDB1 expression in primary macrophages at 72 and 96 hours post infection with a lentivirus vector expressing a DDB1 shRNA. Control cells were infected with a non shRNA expressing lentivirus vector. (B) SIV cDNA and HIV-1 cDNA levels in SIV and HIV-1 infected macrophages 96 h after transduction with lentivirus vectors expressing a DDB1 shRNA or 96 h after transduction with a vector control. Infections done in the presence of AZT were used to assess the level of carry over viral DNA contamination. (0.23 MB TIF) [file ppat.1000057.s003.tif]
